# Primary Prevention of Cardiovascular Disease in Women

**DOI:** 10.14797/mdcvj.1313

**Published:** 2024-03-14

**Authors:** Izza Shahid, Eleonora Avenatti, Anoop Titus, Sadeer Al-Kindi, Khurram Nasir

**Affiliations:** 1 Houston Methodist Academic Institute, Houston, Texas, US; 2 Houston Methodist DeBakey Heart & Vascular Center, Houston, Texas, US

**Keywords:** cardiovascular prevention, women’s health, gender disparities, equity

## Abstract

Cardiovascular disease (CVD) remains a leading cause of mortality in women, necessitating innovative primary prevention strategies. Contemporary guidelines on primary prevention of CVD highlight the increasing prevalence of CVD risk factors and emphasize the significance of female-specific risk enhancers that substantially augment the future risk of CVD. These risk factors occur throughout a woman’s life cycle, such as hormonal contraception, hypertensive disorders of pregnancy, and menopause, all of which confer an added layer of risk in women beyond the conventional risk factors. Despite this, current methods may not fully capture the nuanced vulnerabilities in women that increase their risk of CVD. In this review, we highlight gender-specific risk enhancers and subsequent prevention as well as strategies to improve primary prevention of CVD in women.

## Introduction

Cardiovascular disease (CVD) continues to be a leading cause of mortality among both men and women in the US, with women accounting for up to 420,164 deaths in 2018.^1^ Historically, CVD has predominantly been perceived through a male-centric lens, often leading to a generalized approach in prevention and treatment strategies.^2^ However, sex-specific variations exist in the pathophysiology, symptoms, and treatment efficacy across both genders, thereby warranting a tailored approach that meticulously considers the unique risk factors and clinical presentations that are characteristic of the female population.^3,4^

Recent estimates suggest a decreasing trend of cardiovascular mortality over time. However, a discernible increase in cardiovascular risk factors such as diabetes mellitus (DM) and obesity has been observed in recent years, with estimated projections suggesting DM prevalence in women to increase from 199 million to 313 million by 2040.^5^ This upward trajectory necessitates a focus on primary prevention strategies to help decrease the potential impact of these risk factors on women’s cardiovascular health.^6^ Despite the importance of primary prevention in mitigating future CVD risk, women are underrepresented in trials of preventive therapies such as lipid-lowering drugs and are more likely to be underestimated for their CVD risk.^7^ A nuanced understanding of gender-specific considerations, including biological and systemic factors, and implementation of primary prevention strategies is pivotal to help decrease the future risk of CVD in women. Therefore, in this review we highlight gender-specific risk enhancers and subsequent prevention and discuss strategies to improve primary prevention of CVD in women.

## Gender-specific Risk Factors in Women

### Polycystic Ovarian Syndrome

Polycystic Ovarian Syndrome (PCOS) is prevalent among young women and is characterized by hormonal imbalance and irregular menstrual cycles.^8^ Women with PCOS often encounter a spectrum of metabolic anomalies such as insulin resistance, obesity, and an unfavorable cardiovascular risk profile, predisposing them to an elevated risk of CVD and premature atherosclerosis.^8^ Women with PCOS are estimated to have up to 29% increased risk of cardiovascular events compared with women without PCOS, with population-based studies suggesting up to 4.8% women with PCOS developing incident CVD.^9,10^

To reduce the risk of CVD in women with PCOS, it is essential to adopt a proactive strategy for managing the associated risk factors (Table 1). Regular monitoring and early intervention directed at controlling blood pressure (BP), body mass index (BMI), and lipid levels is essential.^6^ Screening guidelines for evaluating CVD risk in women with PCOS recommend measurement of weight and BMI every 6 to 12 months and measurement of BP at least once per year.^11^ Additionally, screening tests like oral glucose tolerance, fasting glucose, and hemoglobin A1c levels should also be conducted as necessary to evaluate the risk of DM. Lifestyle modifications, including a well-balanced diet and regular physical activity can help assist in mitigating obesity and enhancing insulin sensitivity.

**Table 1:** Select gender-specific risk factors for cardiovascular disease in women and potential primary prevention strategies. BP: blood pressure; BMI: Body Mass Index; CV: cardiovascular: HbA1c: hemoglobin A1c, also called the glycated hemoglobin test


RISK FACTOR	MANAGEMENT

**Polycystic ovarian syndrome**	Regular monitoring of BP, BMI, lipids Screening tests include oral glucose tolerance test, HbA1c Lifestyle modifications

**Hormone contraceptive use**	Contraceptive counselling Evaluate baseline CV risk Reduction of other CV risk factors such as hypertension, obesity

**Fertility therapy**	Evaluate baseline CV risk Adequate counselling for women at potential risk Routine monitoring in a preventive cardiology clinic for surveillance of cardiovascular risk factors

**Hypertensive disorders of pregnancy**	Preconception counselling Aspirin for primary prevention in women at risk of preeclampsia Routine monitoring post birth

**Postmenopausal hormone replacement therapies**	Personalized approach considering individual risk profile and shared decision making

**Breast cancer treatment**	Routine monitoring with a cardio-oncology specialist to monitor potential risk of cardiotoxicity


### Hormonal Contraceptive Use

Combined hormonal oral contraceptives (OCPs), primarily the combination of estrogen and progestin, are widely utilized by women of childbearing age as the contraception of choice in family planning.^12^ Within the US, an estimated 27.7% women of reproductive age report using products that contain exogenous estrogen as hormonal contraceptives.^12^ Although young women are at a lower cardiovascular risk due to increased estrogen receptor expression in their arteries compared with men, which helps regulate arterial tone and reduce arterial remodeling and BP, the use of hormonal contraceptives incurs a nuanced risk concerning cardiovascular health, specifically an elevated risk of thrombotic stroke or myocardial infarction.^13^ In women without known prothrombotic conditions, use of OCPs increases the risk of venous thromboembolism from 2 to 10 per 100,000 to 7 to 10 per 100,000.^14^ This risk also escalates with certain risk factors such as age, smoking, increased BMI, and the presence of hypercoagulable states. In addition, it is dose-dependent, with higher estrogen doses (> 50 micrograms) attributing to an increased risk.^15^ Therefore, careful consideration and strategy are essential when prescribing hormonal contraceptives.

To prevent the risk of CVD in these women, it is crucial to adopt an informed and personalized approach in contraceptive counseling. Healthcare professionals should emphasize mitigating other cardiovascular risk factors, such as hypertension and obesity, and consider the overall cardiovascular profile of the woman. For those at higher cardiovascular risk, a combination of levonorgestrel and 30 microgram of estrogen, progestin-only pills, or intrauterine devices might be more suitable and safer options.^13^

### Fertility Therapy

Fertility therapies, such as in vitro fertilization and intrauterine insemination, have become increasingly prevalent in assisting women with infertility.^16^ Although safe and instrumental in enhancing pregnancy chances, these treatments carry inherent risk associated with adverse cardiovascular events.^17^ Multiple cycles of ovarian hyperstimulation coupled with elevated estrogen levels contribute to a prothrombotic state and promote endothelial injury.^17^ A Swedish population-based study of 23,498 women with live births following in vitro fertilization revealed increased rates of hypertension and a trend towards higher incidence of stroke in women who received fertility therapy compared with women who did not.^18^ Moreover, women who undergo multiple fertility cycles or who fail fertility therapy are also at a heightened risk of CVD. Notably, fertility therapy failure is associated with a 19% increased risk of further adverse cardiovascular events.^19^

To mitigate future adverse events in this subset of patients, women undergoing fertility therapy or those whose therapy was unsuccessful should be routinely monitored in a preventive cardiology clinic for surveillance of cardiovascular risk factors and morbidity. Prior to starting fertility therapy, women should be evaluated for their baseline cardiovascular risk. Healthcare professionals should ensure that these women, especially those with existing comorbidities, are well-informed about the potential risks. This vigilant approach, coupled with ongoing research into preventive strategies can aid in ameliorating potential adverse cardiovascular outcomes in women.

### Hypertensive Disorders of Pregnancy

Hypertensive disorders of pregnancy (HDP), including chronic hypertension, gestational hypertension, preeclampsia, and eclampsia, are marked by hypertension before (chronic hypertension) or after 20 weeks of gestation (gestational hypertension) and are crucial indicators of increased cardiovascular risk later in life.^20,21^ Hypertension occurs in approximately 10% of pregnancies and is one of the leading causes of maternal and fetal mortality.^22^ Among women without prior hypertension, HDP do not only increase the likelihood of developing hypertension post-pregnancy but also accelerate the onset.^23,24,25^ Research suggests that women with a history of HDP tend to be diagnosed with hypertension approximately a decade earlier compared with women who had pregnancies without HDP.^25^

The pathophysiology and consequent development of HDP varies across the specific HDP subtypes. For example, gestational hypertension is defined as the development of hypertension on two separate occasions after 20 weeks of gestation with no evidence of proteinuria.^26^ Pre-eclampsia is distinctively marked by the onset of hypertension and either proteinuria or significant end-organ dysfunction after 20 weeks of gestation.^26^ In contrast, chronic hypertension involves pre-existent hypertension before 20 weeks of gestation and is defined by BP ≥ 140 mm Hg systolic and/or 90 mm Hg diastolic before pregnancy or before 20 weeks of gestation.^26^ The pathophysiology of preeclampsia involves placental ischemia and increased antiangiogenic factors, which leads to decreased uteroplacental perfusion and maternal endothelial damage.^27^ Consequently, this contributes to end-organ hypoperfusion. Prior studies have demonstrated preeclampsia to be associated with an increased risk of mortality, heart failure (HF), ischemic heart disease and stroke, with the CHAMPS (Cardiovascular Health After Maternal Placental Syndrome) study observing a 12-fold increase in CVD risk in women with a history of preeclampsia and metabolic syndrome.^28,29^ Similarly, chronic hypertension is associated with 5- to 10-times increased risk of maternal mortality, heart failure, stroke or acute kidney injury.^30,31^

Given that HDP and CVD share similar risk factors such as obesity, prior hypertension, dyslipidemia, and insulin resistance, strategies aimed at decreasing the risk of developing HDP should involve comprehensive cardiovascular risk assessments, particularly focusing on BP and DM.^27^ Emphasis should also be placed on preconception counselling, including patient education and promoting lifestyle adjustments aimed at reducing cardiovascular risk. Exercise may reduce the risk of gestational hypertension and preeclampsia risk by approximately 30% and 40%, respectively.^32,33^ Following the recent Chronic Hypertension and Pregnancy (CHAP) Study, the American College of Obstetrics and Gynecology (ACOG) recommends initiation or titration of antihypertensive therapy using BP of 140/90 mm Hg as the threshold among pregnant women with chronic hypertension as opposed to the previously recommended BP threshold of 160/110 mm Hg.^26^ Although the management of chronic hypertension in women of childbearing age seeking a pregnancy and pregnant patients is beyond the scope of the present article, it is critical to acknowledge the need to provide appropriate counseling and consider safety of medications and their teratogenic potential. Among women with a history of preeclampsia and preterm delivery or for women with more than one pregnancy complicated by preeclampsia, ACOG recommends daily low-dose aspirin.^34^ Notably, interdisciplinary collaboration involving obstetrics, cardiology, and primary care is essential for enhancing the delivery of preventive care, facilitating a more integrated and effective approach in managing HDP.

### Postmenopausal Hormone Replacement Therapies

Post-menopausal women are at an elevated risk of developing CVD, predominantly due to a decline in endogenous estrogen levels.^35^ Numerous studies suggest that estrogen aids in preventing cardiomyocyte apoptosis.^36^ Although earlier observational studies demonstrated cardioprotective benefits of hormone replacement therapies (HRT), key clinical trials such as the Women’s Health Initiative (WHI) did not conclusively affirm the cardiovascular benefits of menopausal HRTs.^37,38^ In contrast, the 2015 Cochrane Database analysis showed that postmenopausal HRT use was associated with a 24% increased risk of stroke, venous thromboembolism (RR 1.92, 95% CI, 1.36-2.69), and pulmonary embolism (RR 1.81, 95% CI, 1.32-2.48).^39^ Consequently, HRT is not recommended for primary or secondary prevention of CVD, with the US Preventive Services Task Force suggesting that menopausal HRT is neither beneficial nor indicated for preventing the risk of CVD.^40^

Despite this, many women experience severe menopausal symptoms such as osteoporosis, vasomotor symptoms, and sleep disturbances for which HRT remains the most effective treatment. For these patients, a careful evaluation of personalized cardiovascular risk assessment with tailored preventive strategies, treatment benefits, and personal preference is mandatory. The American College of Cardiology (ACC) and American Heart Association (AHA) recommends utilizing the atherosclerotic cardiovascular disease (ASCVD) Pooled Cohort Equation Risk Calculator for ascertaining 10-year CVD risk of women.^41^ HRT should be avoided in women with known CVD, clotting disorder, breast cancer, or if 10-year ASCVD risk is ≥ 7.5%. Caution should be practiced while prescribing HRT in women with known CVD risk factors or if 10-year ASCVD risk is ≥ 5% to 7.4%. However, women who have recent menopause (less than 10 years) and whose 10-year ASCVD risk is < 5% are at a lower risk of HRT-induced adverse CVD effects and therefore HRT can be recommended.^41^ It is important to note that HRT initiated after 10 years of menopause or in patients > 60 years of age portends greater absolute risk of CVD, stroke, and thromboembolism with fewer clinical benefits.^41^ Therefore, utilization of HRT should be meticulously tailored to the individual’s unique clinical profile, risk factors, and preferences.

### Breast Cancer Treatment

Breast cancer treatment has markedly improved in the recent decade, leading to improved survival following a breast cancer diagnosis.^42^ Although the availability of novel therapeutic regimens such as anthracyclines and trastuzumab have significantly improved survival outcomes in patients with breast cancer, these treatments come with their own challenges, particularly an increased susceptibility to adverse cardiovascular events.^43^ Anthracyclines cause cardiotoxicity and lead to gradual decline in left ventricular ejection fraction (LVEF).^43^ Studies suggest that a moderate yet continuous reduction of 4% in LVEF persists even 3 years following exposure to anthracyclines.^44^ Similarly, trastuzumab, primarily used in managing HER2-positive breast cancers, also contributes to cardiac dysfunction especially when administered alongside anthracyclines.^45^ Moreover, breast radiation therapy can cause constrictive pericarditis, myocardial fibrosis, and coronary artery lesions.^46^

Given that breast cancer survivors have an increased risk of death from cardiovascular causes, which becomes more pronounced 7 to 8 years after their initial diagnosis, a thoughtful integration of preventive cardiology into the care of breast cancer survivors is important.^47^ Routine monitoring and assessments in a preventive cardiology clinic can be instrumental in the early identification and management of cardiovascular risk factors. Evidence suggests that use of cardioprotective agents such as angiotensin converting enzyme inhibitors (ACEIs), beta-blockers, mineralocorticoid receptor antagonists, and statins in women undergoing anthracycline-trastuzumab chemotherapy for breast cancer can reduce the future risk of cardiotoxicity.^48,49^ For example, results from a meta-analysis including seven observational studies (N = 2,262 cancer patients) on statin use in patients with breast cancer demonstrated a significantly reduced risk of cardiotoxicity (RR 0.45, 95% CI, 0.29-0.70; defined as incidence of HF or a ≥10% decline in LVEF from a baseline value to an absolute value of < 55%).^48^ Such proactive strategies can potentially allow for the timely modification of treatment plans, accommodating the cardiovascular needs of the patient without compromising the efficacy of cancer treatment.

## Current Guidelines for Risk Stratification

The 2019 ACC/AHA guidelines recommend utilizing a 10-year ASCVD risk evaluation that is estimated by the race- and sex-specific pooled cohort equations for adults aged 40 to 75 years.^20^ These ASCVD risk assessment tools have the potential to underestimate and/or overestimate cardiovascular risk in women as these were based on traditional risk factors and older cohorts. Due to these limitations, the ACC/AHA guidelines recommend adding risk-enhancing factors to modify the ASCVD estimate among adults at borderline (5% to < 7.5%) and intermediate (≥ 7.5% to < 20%) risk.^20^ Notably, these guidelines now recommend adding female-specific risk enhancers such as history of premature menopause and preeclampsia.^20^

If uncertainty persists regarding ASCVD risk and the net benefit of initiating preventive therapy, guidelines recommend using coronary artery calcium (CAC) by non-contrast CT to better ascertain the ASCVD risk.^20^ CAC has proven to be effective in predicting cardiovascular risk independent of sex and improves risk prediction among women who may have been categorized as low risk by traditional risk scoring tools.^50,51^

## Strategies to Improve Cardiovascular Prevention

### Pharmacological Interventions

#### Statins

**Figure 1:**
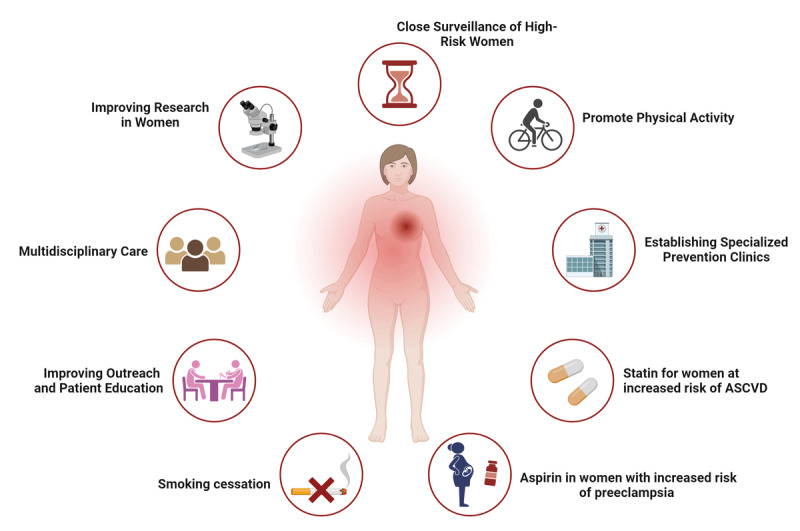
Strategies to improve primary prevention of cardiovascular disease in women. ASCVD: atherosclerotic cardiovascular disease

Cardiovascular prevention strategies have traditionally centered around lipid-lowering therapy due to substantial evidence supporting the use of medications such as statins for ASCVD prevention (Figure 1). Statins are uniformly effective in both men and women, and current recommendations do not differentiate based on sex in endorsing statin use for primary prevention in patients at increased ASCVD risk.^20,52,53^ The 2019 ACC/AHA Guidelines on the Primary Prevention of CVD indicate the use of statins for patients with clinical ASCVD, severe hypercholesterolemia, DM mellitus in adults (aged 45-70 years), or for primary prevention in adults aged 40 to 75 years who are at a heightened risk of ASCVD. This includes individuals with a risk estimation of 20% or higher, adults with intermediate risk (ranging from 7.5% to < 20%) or borderline risk (5% to < 7.5%). However, current guidelines, while universal in their application, do underscore the necessity to incorporate gender-specific risk factors such as preeclampsia and early menopause for a more nuanced risk stratification in women.^53^

Despite this, an evident disparity persists in the adherence to statin therapies between genders. The use of statins remains suboptimal among women, who are significantly less likely to initiate and maintain statin treatment compared to their male counterparts.^54^ Data from the National Health and Nutrition Examination Survey revealed worse dyslipidemia control in women without underlying CVD compared to men, a trend that was observed to intensify over time.^55^ Similar trends are observed in Europe.^56^ The reasons for these disparities are multifactorial and mostly attributed to systemic gender disparities in health care, marked by decreased awareness of cardiovascular risks in women and historical underrepresentation of women in clinical trials.^54,57^

Additionally, medication intolerance and side effects might be more common in women who may exhibit heightened sensitivity to statin side effects, necessitating a more tailored approach.^58^ Concerns regarding statin use among women of childbearing age and during pregnancy further complicate adherence due to lack of safety data during pregnancy. These women should be counseled on the opportunity to discontinue statin treatment prior to a planned pregnancy. However, flexibility exists, with agencies such as the US Food and Drug Administration revising their safety guidelines to adopt a more patient-centered stance for women at very high ASCVD risk.^59^ Encouraging evidence exists regarding the use of pravastatin, which has shown to be safe and effective in preventing preeclampsia in very high-risk patients.^60^

#### Other Lipid-Lowering Therapies

Non-statin treatments present an alternative to statins to manage hyperlipidemia, although data on their efficacy in primary prevention overall and specifically in women is limited. The IMPROVE IT (Improved Reduction of Outcomes: Vytorin Efficacy International Trial) study, for example, established efficacy of ezetimibe as a lipid-lowering agent on top of statin treatment, but it enrolled only 25% women and was a secondary prevention trial.^61^ The newer class of PCSK9i inhibitors, which are potent lipid-lowering agents, has not been investigated in primary prevention trials, and the two pivotal studies that demonstrated their efficacy in secondary prevention had a significant gender gap (women < 25%).^62,63^ On the contrary, the CLEAR (Cholesterol Lowering via Bempedoic Acid [ECT1002], an ACL-Inhibiting Regimen) Outcomes trial enrolled 48% of women in its study population, and 30% of the patients were enrolled for primary prevention, making a compelling case for the potential use of bempedoic acid in this space.^64^

#### Antihypertensives

Hypertension is one of the most common and modifiable risk factors for ASCVD. Appropriate screening and identification of patients in need of adequate management is pivotal, as is ensuring that treatment goals are obtained with pharmacological and nonpharmacological approaches. Current recommendations by the AHA/ACC suggest achieving a BP goal < 130/80 mm Hg in subjects who are at high risk (> 10%) for ASCVD based on the pooled cohort equations. Pharmacological treatment of hypertension can reduce morbidity and mortality irrespective of gender.^65^ Although pathophysiological differences exist in the development of hypertension and its complications between men and women, no gender differences exist in treatment among any major drug classes currently recommended for hypertension treatment.^66,67^ Hence, there are no recommendations for a differential approach to treatment based on gender, with a few caveats. The major notable exception regards pharmacological treatment for women of childbearing age given the safety profile of some of these medications. ACEIs and/or angiotensin receptor blockers are contraindicated in pregnancy and should not be prescribed in women of childbearing age without an appropriate patient-centric discussion. An algorithm for remote treatment of hypertension in women of childbearing age has been developed and successfully implemented with the use of calcium channel blockers and labetalol.^68^ Management of hypertension during pregnancy has recently been addressed by ACOG. A treatment threshold for BP values of 140/90 mm Hg is currently recommended based on the CHAP trial.^69^

#### Aspirin in Primary Prevention

Recommendations on the use of aspirin for primary prevention have significantly evolved in the recent decade based on contemporary clinical trial evidence. The multicenter ASCEND (A Study of Cardiovascular Events iN Diabetes), ARRIVE (A Randomized Trial of Induction Versus Expectant Management), and ASPREE (Aspirin in Reducing Events in the Elderly) trials were concordant in finding a lack of net benefit for the use of daily aspirin in primary prevention of ASCVD.^70,71,72^ Notably, the percentage of women enrolled ranged from 30% (ARRIVE) to 56% (ASPREE). Recognizing such lack of benefit and the bleeding risk associated with aspirin use, the 2019 ACC/AHA prevention guidelines recommend against the use of aspirin for primary prevention in patients > 70 years old and in patients with prior bleeding or at risk for bleeding, with no gender-specific recommendations. A personalized approach centered around evaluation of the patient’s individual risk and benefits is encouraged for determining the appropriateness of aspirin use in preventive strategies. Key considerations for recommending aspirin in patients with low bleeding risk include the presence of specific risk enhancers such as current smoking habits, substantial subclinical atherosclerosis (evidenced by a CAC score > 100) and a strong family history of ASCVD.^20^ Other areas include utilization of low-dose aspirin in pregnant women at high risk of pre-eclampsia.^34^

### Lifestyle Modifications

Primary prevention of CVD focuses on initiation and maintenance of healthy lifestyle habits that encompass adequate levels of physical activity, healthy eating patterns, normal BMI, and sleep hygiene. The ACC/AHA guidelines and US government agencies recommend a goal of 150 minutes of moderate intensity activity (or 75 minutes of high intensity activity) weekly.^73^ The physical activity should comprise of aerobic exercise alongside some strength or resistance training preferably twice a week. Unfortunately, in a recent survey following the release of the US Department of Health and Human Services guidelines, only 1 in 10 responders, independent of gender, were aware of such guidelines and recommendations.^74^ The majority of Americans, particularly women, do not reach recommended goals, with variations observed across racial and ethnic groups.^75^ Among women, the most commonly reported reasons for physical inactivity include lack of time and support for competing responsibilities (eg, childcare) as well as lack of places to exercise.^76^ However, social determinants of health, cultural factors, and gender disparities in the division of labor likely remain additional crucial factors.

Dietary choices play a pivotal role in primary prevention strategies for ASCVD. A substantial body of evidence robustly supports the Mediterranean diet as a particularly effective dietary regimen in mitigating ASCVD risks. Characterized by a preference for whole foods and reduced reliance on processed and ultra-processed options, the Mediterranean diet is abundant in nuts, olive oil, legumes, fish, and white meats.^77^ Adherence to healthy dietary patterns appears to be consistent across all genders. However, it is noteworthy that women, following a diagnosis of CVD, seem to exhibit enhanced adherence to healthier dietary practices, as observed in findings from the PURE (Prospective Urban Rural Epidemiology) study.^78^

### Systemic and Community-Level Strategies

#### Establishing Specialized Cardiometabolic and Preventive Medicine Clinics

**Figure 2:**
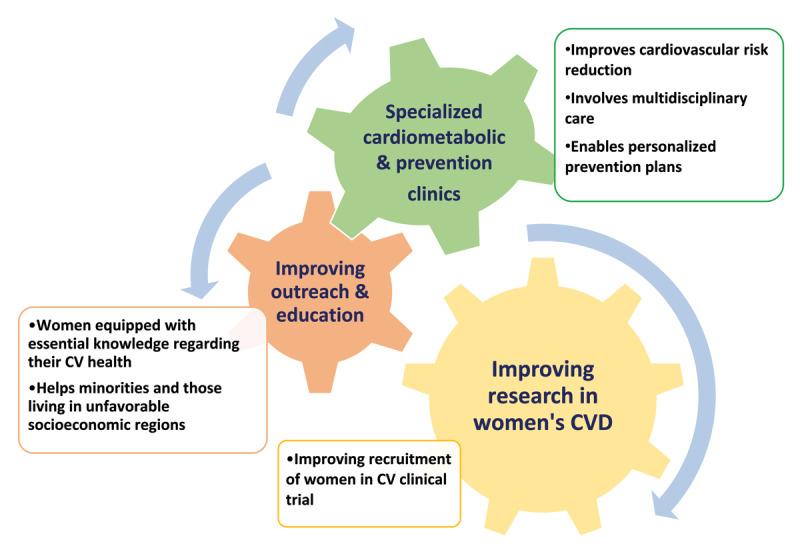
Systemic and community-level strategies for primary prevention of cardiovascular disease in women. CV: cardiovascular; CVD: cardiovascular disease

Prevention and Cardiometabolic Clinics (PCMCs) emphasize risk factor mitigation at both the primary and secondary levels of prevention. Studies focusing on the impact of PCMCs have shown better low-density lipoprotein concentration, total cholesterol, and weight reduction compared with patients enrolled in general or interventional cardiology clinics (Figure 2).^79^ For example, a 6-month prospective cohort study demonstrated improved achievement of BP targets (from 69.2% to 80.5%) among women with a prior history of HDP who attended a multidisciplinary Women’s Heart Clinic.^80^ This translates to improved CVD risk reduction in the population. PCMCs integrate the practice of an endocrinologist, nephrologist, and women and men’s health to general cardiology that potentially reduces patient burnout. Dedicated clinics such as these would provide precision care beyond the scope of the guidelines by using current evidence of the latest trials. A highly effective strategy to bridge the gap between emerging evidence and clinical practice includes checklist methods for ASCVD prevention.^81^ Importantly, utilization of PCMCs can aid in overcoming therapeutic inertia in the use of screening tests such as CAC, which allows for better screening, early identification of at-risk patients, reclassification, and adequate resource allocation.^82^ This is particularly useful for women, who are often perceived as having a lower CVD risk compared with men. Utilization of PCMCs focused on women’s health allows for evaluation of baseline cardiovascular risk and aids in the implementation of personalized prevention plans that may differ depending on specific risk-enhancing factors such as obstetric and gynecological history, history of cancer, and family history of CVD.^83^

#### Improving Outreach and Education

Improving outreach and education specifically tailored to women is paramount in enhancing the primary prevention strategies against CVD. A nuanced, gender-specific educational approach ensures that women are equipped with the essential knowledge and resources to navigate and mitigate their unique cardiovascular risks effectively. Prior studies show that outreach has improved patient-physician discussion regarding risk factors and statin prescription.^84^ Such interventions would be notably important in minority races, the uninsured, and individuals living in lower socioeconomic areas where use of cardiovascular prevention strategies remain low.^85^ With the help of community health workers, outreach has also uncovered at-risk populations through screening.^86^ Educating these populations can facilitate deeper comprehension of the significance of regular health screenings and timely medical consultations. Therefore, empowering women with comprehensive and accessible information promotes them towards making informed, proactive decisions regarding their cardiovascular well-being.

#### Incorporating Gender-Specific Research

Despite the increased prevalence of CVD, women remain underrepresented in cardiometabolic drug trials.^57^ This leads to a lack of understanding of differential effectiveness and potential risks associated with various cardiometabolic drugs in women. This consequently impacts the accuracy and applicability of primary prevention guidelines, potentially leading to less optimized therapeutic strategies for women. In addition, the availability of limited gender-specific insights could compromise the efficacy of pharmacological interventions, thereby limiting the advancement of tailored prevention approaches that consider women’s distinct biological and hormonal considerations. To bridge this gap, concerted efforts are required to improve enrollment of women in clinical trials, which can potentially be achieved by improving diversity in clinical trial leadership and enhanced funding.

## Conclusion

The enduring challenge of CVD in women necessitates a multifaceted and nuanced approach to primary prevention. It is important to understand the critical role of gender-specific risk enhancers and their intricate interplay in influencing CVD risk among women. These include biological considerations that span throughout a woman’s life cycle, including stages such as the reproductive age, pregnancy, and the postmenopausal period. For women at high risk of ASCVD, strategies such as use of statin therapy is recommended. To improve implementation of primary prevention strategies, dedicated PCMCs should be established where high-risk women can be routinely monitored. Furthermore, outreach, education, awareness, and inclusion of women in clinical trials can help provide a robust evidence base that is both inclusive and reflective of the multifaceted nature of CVD risk among women.

## Key Points

- Cardiovascular disease (CVD) in women presents unique challenges and risk factors that necessitate gender-specific primary prevention strategies.
- Systemic, community-based strategies and pharmacological interventions are key in primary prevention of CVD in women.
- The implementation of specialized Prevention and Cardiometabolic Clinics can facilitate personalized care and routine monitoring in women.
- Enhancing outreach and education efforts is essential for improving awareness and the adoption of preventive measures against CVD.
- Statins remain the cornerstone of pharmacological prevention but must be prescribed within a framework that accounts for the distinct physiological responses and life stages of women.
- Systemic changes, including the improvement of gender-specific research and the inclusion of female-centric data in guidelines, are vital for advancing primary prevention efforts and reducing the burden of CVD in women.

## CME Credit Opportunity

Houston Methodist is accredited by the Accreditation Council for Continuing Medical Education (ACCME) to provide continuing medical education for physicians.

Houston Methodist designates this Journal-based CME activity for a maximum of *1 AMA PRA Category 1 Credit*™. Physicians should claim only the credit commensurate with the extent of their participation in the activity.

Click to earn CME credit: learn.houstonmethodist.org/MDCVJ-20.2.
